# scFv biofunctionalized nanoparticles to effective and safe targeting of CEA-expressing colorectal cancer cells

**DOI:** 10.1186/s12951-023-02126-4

**Published:** 2023-10-02

**Authors:** Maria José Silveira, Cláudia Martins, Tânia Cruz, Flávia Castro, Ângela Amorim-Costa, Kerry Chester, Maria José Oliveira, Bruno Sarmento

**Affiliations:** 1 i3S – Instituto de Investigação e Inovação em Saúde, Universidade do Porto, Rua Alfredo Allen 208, 4200-135 Porto, Portugal; 2 ICBAS – Instituto de Ciências Biomédicas Abel Salazar, Universidade do Porto, Rua de Jorge Viterbo Ferreira 228, 4050-313 Porto, Portugal; 3 UCL – University College London Cancer Institute, London, UK; 4 FMUP – Faculdade de Medicina, Universidade do Porto, Alameda Prof. Hernâni Monteiro, 4200-319 Porto, Portugal; 5 IUCS-CESPU, Rua Central de Gandra 1317, 4585-116 Gandra, Portugal

**Keywords:** Cancer therapy, Carcinoembryonic antigen, Drug delivery, Nanomedicine, Single-chain variable fragment functionalization, Targeted therapies

## Abstract

Colorectal cancer (CRC) is one of the deadliest cancers worldwide, with the 5 year survival rate in metastatic cases limited to 12%. The design of targeted and effective therapeutics remains a major unmet clinical need in CRC treatment. Carcinoembryonic antigen (CEA), a glycoprotein overexpressed in most colorectal tumors, may constitute a promising molecule for generating novel CEA-targeted therapeutic strategies for CRC treatment. Here, we developed a smart nanoplatform based on chemical conjugation of an anti-CEA single-chain variable fragment (scFv), MFE-23, with PLGA-PEG polymers to deliver the standard 5-Fluorouracil (5-FU) chemotherapy to CRC cells. We confirmed the specificity of the developed CEA-targeted NPs on the internalization by CEA-expressing CRC cells, with an enhance of threefold in the cell uptake. Additionally, CEA-targeted NPs loaded with 5-FU induced higher cytotoxicity in CEA-expressing cells, after 24 h and 48 h of treatment, reinforcing the specificity of the targeted NPs. Lastly, the safety of CEA-targeted NPs loaded with 5-FU was evaluated in donor-isolated macrophages, with no relevant impact on their metabolic activity nor polarization. Altogether, this proof of concept supports the CEA-mediated internalization of targeted NPs as a promising chemotherapeutic strategy for further investigation in different CEA-associated cancers and respective metastatic sites.Authors: Please confirm if the author names are presented accurately and in the correct sequence (given name, middle name/initial, family name). Author 1 Given name: [Maria José] Last name [Silveira]. Author 7 Given name: [Maria José] Last name [Oliveira]. Also, kindly confirm the details in the metadata are correctokAffiliations: Please check and confirm that the authors and their respective affiliations have been correctly identified and amend if necessary.ok

**Graphical Abstract:**

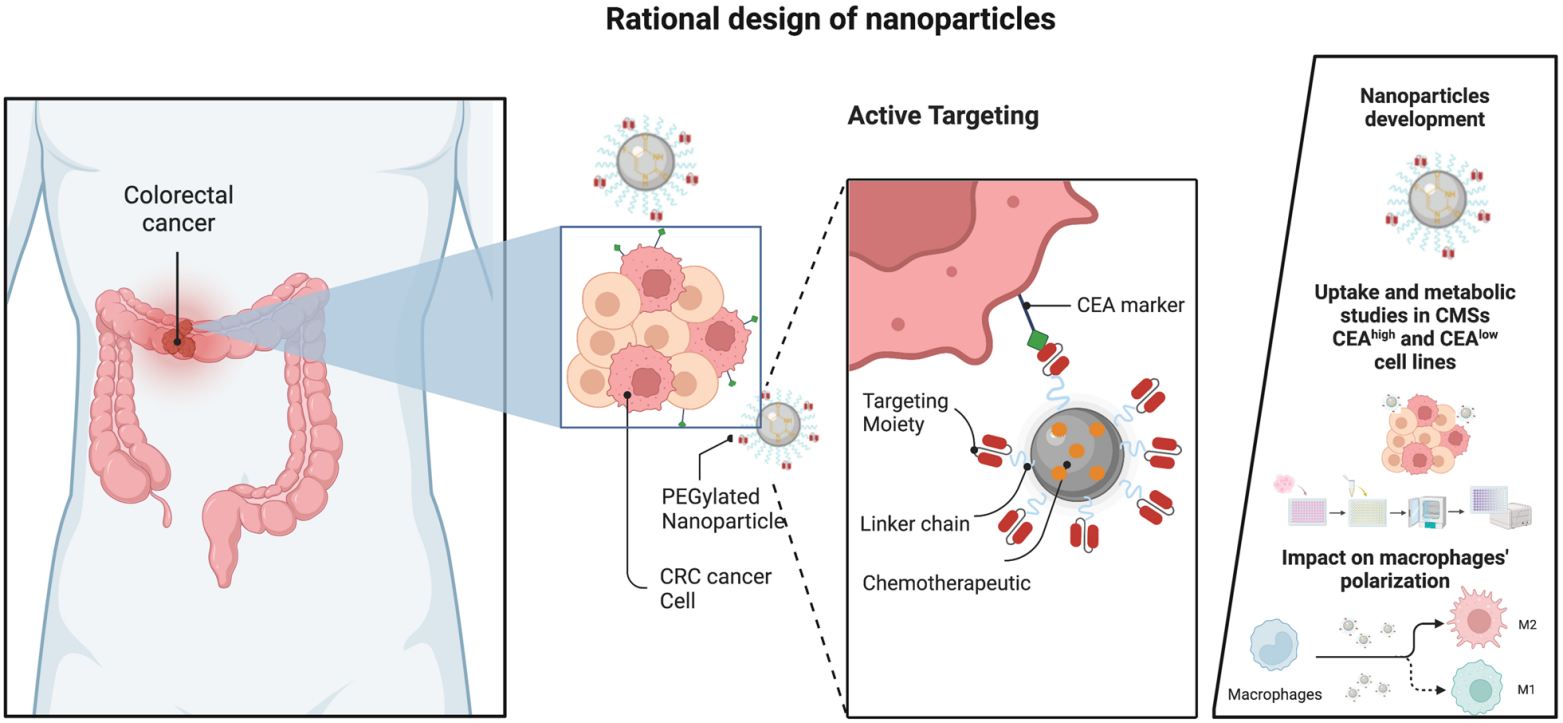

**Supplementary Information:**

The online version contains supplementary material available at 10.1186/s12951-023-02126-4.

## Background

Colorectal cancer (CRC) is the second most lethal cancer worldwide with around 12% of 5 year survival rate in metastatic cases [1, 2]. The molecular mechanisms of CRC, associated microbiota, epigenetic and genetic alterations, as well as its tumor microenvironment (TME), confer highly intertumoral and intratumor heterogenicity. Such complexity renders CRC treatment particularly challenging and results in numerous cases of drug resistance and tumor relapse [3]. Having in consideration the aforementioned features of CRC, the stratification into four consensus molecular subtypes (CMSs) reflects the molecular and genetic profiles as well as immune infiltrate differences among these tumors [4]. In fact, the CMSs classification brought a new paradigm to the CRC treatment, allocating microsatellite instable (MSI) or microsatellite stable (MSS) tumors into different subtypes, allowing a better selection of therapy and its response prediction [5].Article structure: Please confirm the section headings are correctly identified.ok

The fluoropyrimidine, 5-Fluorouracil (5-FU), an antimetabolite with anticancer effects, remains a mainstream option of chemotherapy to treat high-risk stage II, stage III and IV CRC patients, as single-agent therapy, and multiple-agent regimens (FOLFOX, FOLFIRI, XELOX, and SOX) [6]. However, despite multiple benefits, 5-FU-based therapies still hold very low response rates, are frequently associated with chemoresistance, low bioavailability, low drug uptake, lack of tumor targetability and high systemic toxicity [7]. Specifically, for patient harboring tumors stratified into CMS1 and CMS4 subtypes, 5-FU adjuvant chemotherapy does not seem to improve overall survival (OS) and significantly decreases the 5 year disease-free survival rate from 83.3% to 30.0%. Whereas, in tumors classified into CMS2 and CMS3, 5-FU treatment is associated with increasing levels of OS and 5 year disease-free survival rates [8].

The TME has been reported as a critical factor, affecting CRC therapeutic outcomes and treatment resistance [9]. One of the most important component of the TME are tumor-associated macrophages (TAMs), usually divided into a spectrum of phenotypes between extreme profiles: M1 macrophages (pro-inflammatory) that act as tumor suppressors, and M2 macrophages (anti-inflammatory) which promote cancer progression [10]. In patients treated with 5-FU, the increase of TAMs infiltration has been negatively correlated with tumor growth [11]. Moreover, some authors have investigated the synergistic effect of 5-FU and M1 macrophages whose findings are associated with higher rates of colorectal cell death [12, 13]. However, when TAMs infiltration is enriched with M2 macrophages, they confer CRC resistance to 5-FU, impairing patients OS [14]. Accordingly, the reported limitations in the therapeutic efficacy of 5-FU in regarding its short half-life, high cytotoxicity, macrophages polarization and low bioavailability, elucidate the need for advanced drug delivery systems as potential tools to enhance 5-FU pharmacokinetic properties and pharmacological performance.

Recently, nanotechnology-based strategies have been emerging as promising strategies to revolutionize illness treatments, with remarkable findings in the way how drugs and molecules are delivered and released [15]. Different nanotechnology-based strategies have been explored for promoting delivery of 5-FU to tumors [16]. However, efforts are required to improve pharmacokinetic aspects and accelerate translation to the clinic [17] Particularly, PEGylated nanoparticles (NPs) based on the poly (lactic-co-glycolic acid) (PLGA) polymer, have gained significant interest due to its biodegradable properties, non-toxicity, and possibility of a targeted cargo delivery by surface modification with targeting moieties directed to the biological site of interest [18, 19].

The carcinoembryonic antigen (CEA) tumor-associated antigen is a membrane-anchored glycoprotein, overexpressed in 90–95% of colorectal primary tumors and respective metastases [20]. Moreover, CEA is frequently used for diagnosis and prognosis of CRC, constituting a promising target molecule for CRC therapy [21]. Therefore, many monoclonal antibodies have been developed against CEA with diagnosis and therapeutic proposes [22, 23]. Recent advantages in antibody engineering have produced different types of antibodies fragments (e.g., single-chain variable (scFv)). Particularly, the MFE-23 scFv has been shown to effectively target CRC in imaging and antibody-directed pro-drug therapy strategies, due to its enhanced tumor cell internalization, prolonged half-life (superior to 4 days at 37 ºC), and presence of a binding domain for anti-CEA immunotoxins [24, 25].

The major goal of this work was to develop CEA-targeted NPs of PLGA and PEG as a novel platform to deliver 5-FU to CRC cells, thus increasing 5-FU bioavailability and providing a tumor cell-specific anti-cancer activity. Here, we described the NPs manufacture process and detailed their physicochemical characterization. Moreover, we assessed the specificity and selectively of CEA-targeted NPs loaded with 5-FU in CMS1 and CMS4 CRC cell lines, as well as their effect on cell metabolic activity inhibition. Lastly, we assessed the potential impact of the developed NPs on the metabolic activity and polarization of isolated from healthy blood donor’s macrophages.

## Methods

To prepare and characterize NPs, PLGA 5004 (50:50 LA: GA; 44 K; Purasorb^®^ PDLG 5004A) was kindly provided by Corbion-Purac biomaterials. PLGA (30 K)-PEG (5 K)-maleimide (PLGA-PEG-Mal), PLGA (30 K)-FITC and PLGA (30 K)-Rhodamine B were purchased from RuixiBiotech^®^; Anti-CEA scFv was kindly developed and provided by Kerry Chester’s group at UCL Cancer Institute; 5-FU, Ammonium Hydroxide (NH4OH) and dichloromethane (DCM) were acquired from Sigma-Aldrich^®^. Tween^®^ 80, sodium chloride, tris[2-carboxyethyl] phosphine (TCEP), and diethyl ether from Sigma-Aldrich^®^; acetonitrile (ACN), Amicon^®^ Ultra-15 centrifugal filter units (molecular weight cutoff of 100 K) were acquired from MERCK^®^ Millipore; dimethylformamide (DMF) and trifluoroacetic acid (TFA) from Acros Organics. XTerra RP-18 column (5 μm, 4.6 × 250 mm) from waters and Zorbax 300SB-C8 Narrow-bore column (5 μm, 2.1 × 150 mm) from Agilent Technologies. Ultrapure water was prepared in-house with a conductivity of 0.055 μS/cm and a resistivity of 18.2 MΩ.cm, using MilliQ^®^ station from Millipore Corporation. For cell cultures, T flasks were acquired from Orange Scientific; Dulbecco's Modified Eagle Medium (DMEM), Roswell Park Memorial Institute (RPMI) 1640 Medium, Trypsin, Fetal Bovine Serum (FBS) and penicillin–streptomycin from Gibco^®^. Accutase from Thermo Fisher Scientific^®^. RosetteSep-Human Monocyte Enrichment Cocktail from StemCell Technologies. Histopaque-1077, Resazurin and Triton X-100 and Lipopolysaccharides from Escherichia coli O111:B4 (LPS) were purchased from Sigma-Aldrich^®^, Macrophage colony-stimulating factor (M-CSF) and Recombinant Human Interleukin-10 (IL-10) obtained from ImmunoTools. Concerning microscopy and flow cytometry staining, CEA Monoclonal Antibody (1106) Alexa Fluor 488-anti mouse, anti-HLA-DR/PB and cell Mask Green Stain (H32714—CS CellMask Green stain^™^) were purchased from Invitrogen. The antibodies anti-CD14/APC and anti-CD86/FITC were obtained from ImmunoTools. The anti-CD206/PE was purchased from BioLegend. Anti-CD163/PE was acquired from BD Biosciences. The anti-PDL1/FITC was acquired from BD-Pharmingen, while the Live/Dead Fixable Viability Dye eFluor^™^ 780 and 4′,6-diamidino-2-phenylindole (DAPI) from Thermo Fisher Scientific^®^.

## Anti-CEA scFv chemical conjugation to the PLGA-PEG-mal polymer

Anti-CEA scFv containing a disulfide bond molecular weight (MW) ≈ 27 K,11.43 mg, 0.42 μmol) was treated with tris(2-carboxyethyl) phosphine (1.5 mg, 13.2 5.23 μmol) in 2 mL anhydrous dimethylformamide and stirred for 1 h at room temperature (RT) under inert atmosphere. 15 mg of PLGA (30 K)-PEG(5 K)-Mal (Mn ≈ 35 K, 0.43 μmol) in 1 mL anhydrous dimethylformamide were then added to the mixture and allowed to react for 24 h at 4 °C under inert atmosphere. The reaction mixture was poured into cold diethyl ether (25 mL), washed first time with 40 mL of miliQ water, then with 20 mL of miliQ water and finally with cold diethyl ether (5 mL). The conjugated polymer (PLGA-PEG-scFv) was then, dried under vacuum, in a vacuum oven overnight at 20ºC.

## Characterization of targeted polymer PLGA-PEG-scFv

^1^H NMR measurements were performed with a BRUKER AVANCE III 600 MHz (Bruker Corporation) spectrometer at 25 °C in deuterated dichloromethane. Chemical shifts are reported in ppm (δ units) and were referenced to the residual solvent signal. Mnova software (Mestrelab Research) was used for analysis processing. The conjugation efficiency (CE) of PLGA-PEG-scFv was then calculated by indirect method using HPLC technique, according to the following equation:

$$CE \left(\%\right)=\frac{Initial \,mass \,of \,scFv-recovered\,\,mass \,of \,scFv}{Initial \,\,mass \,of \,scFv} \times 100$$

Briefly, the amount of scFv in the final conjugated polymer was quantified by indirect method through the difference between the total amount of scFv in the initial reaction and the final amount of scFv detected in the supernatants after the washes, in the purification step of the conjugated polymer, described above. The chromatographic analysis was performed by RP-HPLC with ultraviolet [26] detection using a Hitachi LaChrom Elite^®^ HPLC System (Hitachi High Technologies America, Inc). The RP-C8 column (Zorbax 300SB-C8 Narrow-bore) (5 μm, 2.1 × 150 mm) was used as stationary phase with a mobile phase composed by water with 0.1% of TFA (eluent A) and acetonitrile with 0.1% of TFA (eluent B) in Gradient method. The chromatographic analysis was performed at a flow rate of 0.7 mL/min in a gradient mode started at 30% of eluent B, increasing for to 40% of B in 12 min and kept constant at 40% for B for 3 min. From 15 to 15.1, eluent B decreased to 30% and kept constant until 20 min. The column oven was kept at 25 °C. The absorbance was read at 280 nm. The injection volume was 30 μL.

## Manufacture of 5-FU loaded CEA-targeted nanoparticles

NPs containing 5-FU, Sigma-Aldrich, were generated by a modified water-in-oil-in-water (w/o/w) double emulsion-solvent evaporation technique. Briefly, 20 mg of polymer of PLGA (Mw ≈ 44 K in a 50/50 M ratio of DL-lactide and Glycolide, viscosity midpoint 0.4 dl/g viscosity) was mixed with 10% of PLGA-PEG-scFv and dissolved in 4 mL Dichloromethane (Sigma-Aldrich) for 3 h. Afterwards 0.2 mL of 50 mg/mL 5-FU in 1 N NH4OH was emulsified at 70% amplitude for 30 s using a Vibra-CellTM ultrasonic processor in an ice bath. The first emulsion was then, poured into 12 mL of 2% Tween 80 (MERCK) aqueous solution and sonicated for 60 s, reaching the second emulsion (w/o/w). The final emulsion was then added to 24 mL of the same surfactant. Dichloromethane evaporation from the final solution occurred for 2 h and 30 under magnetic stirring at 600 rpm. Nanoparticles were washed twice with 10 mL of ultrapure water by centrifugation using Amicon^®^ filters of 100 K (Millipore Corporation) at 2500×*g* for 90 min.

## Characterization of nanoparticles

### Average particle size, polydispersity index, surface charge and morphology of nanoparticles

Nanoparticles were characterized for their Z-average and polydispersity index (PDI) by dynamic light scattering (DLS), and zeta-potential (ζ-potential) through laser Doppler anemometry (LDA) using a Malvern Zetasizer Nano ZS instrument (Malvern Instruments Ltd). For these measurements, samples were diluted in an ionic solution of 10 mM sodium chloride (NaCl) at a 0.2 mg/mL particle concentration. Values reported are the mean ± standard deviation (SD) of at least three different batches. The morphological features of NPs were analyzed by transmission electron microscopy (TEM) with a JEOL JEM 1400 microscope (JEOL Ltd) at an accelerating voltage of 120 kV. Briefly, 10 μL of each NPs sample were mounted on nickel grids and left to stain for 2 min approximately.

### 5-FU association efficiency and drug loading

The amount of 5-FU encapsulated into NPs was directly quantified by liquid–liquid phase separation. Briefly, NPs were freeze dried after their purification, and consequently solubilized in 4 mL DCM overnight. In the day after, miliQ water in same proportion was added to the first solution to solubilize 5-FU. The presence of this anti-cancer metabolite, 5-FU, was then analyzed by chromatography using a XTerra-C18 column as stationary phase with a mobile phase composed by water (eluent A) and acetonitrile (eluent B) in an isocratic method. The chromatographic analysis was performed with at a flow rate of 0.7 mL/min using 95:5 (eluent A: eluent B) in 10 min. The column oven was kept at 25 °C. The absorbance was read at 266 nm. The injection volume was 20μL, association efficiency (AE) and drug loading [4] were calculated using the followed equations:

$$AE\boldsymbol{ }\left(\boldsymbol{\%}\right)=\frac{Mass\,\, of \,encapsulated \,5-FU}{inital \,mass\, of\,\, 5-FU} \times 100$$

$$DL(\%)\frac{Mass \,of \,\,encapsulated \,\,5-FU}{Total \,mass \,\,of \,nanoparticles} \times 100$$

### Colloidal stability of nanoparticles

The stability of the developed NPs was evaluated during 31 days at 4 ºC in a timepoints of 0,1,2,3,7 and 31 days. Afterwards, NPs were evaluated by DLS as previously described.

### 5-FU in vitro cumulative release

In vitro release behavior of 5-FU encapsulated into anti-CEA PLGA-PEG NPs was performed at 37 °C under gentle stirring rate (150 rpm) in phosphate buffered saline (PBS) pH 7.4 and evaluated in different time points (0, 0.25, 0.5, 1, 1.5,3, 4,72, 120, 140 and 552 h). The collected samples were centrifuged at 17,000×*g* during 15 min (4 °C), and the supernatant was used for HPLC analysis, as previously described.

## Cell lines and cell culture reagents

All CRC cell lines were obtained from American Type Culture Collection (ATCC). HCT15, HCT116, HT29, RKO and SW480 CRC epithelial cell lines, were cultured in cell culture flasks in RPMI1640 medium (Gibco) with FBS (10% v/v) (Biowest) and penicillin–streptomycin (1% v/v), (Gibco). SW48, Caco-2 and LS174T CRC epithelial cell lines were cultured in DMEM (Gibco), supplemented with FBS, (10% v/v) and penicillin–streptomycin (1% v/v). Cell culture medium was changed every 2–3 days and cell lines were sub-cultured upon trypsin treatment. Cell cultures were kept in an incubator (Cell Culture CO_2_ incubator, ESCO GB Ltd) at 37 °C with 5% CO_2_ and 95% relative humidity. Mycoplasma detection was routinely performed.

### Ethics statement

According to the principles of Declaration of Helsinki, and by the ethical approval of Centro Hospitalar Universitário São João Ethics Committee, protocol reference 90/19, blood donors were informed through a written consent that the products of their blood collections could be used for research purposes. Monocytes were isolated from surplus buffy coats from healthy blood donors, gently provided by the Immunohemotherapy Department of Centro Hospitalar Universitário São João (CHUSJ), Porto, Portugal.

### Human monocytes isolation

As previously reported, human monocytes were isolated from healthy blood donors’ buffy coats [27]. Briefly, buffy coats were centrifuged and incubated with RosetteSep human monocyte enrichment kit (StemCell Technologies) in order to obtain peripheral blood mononuclear cells (PBMCs). After, each sample was diluted 1:1 with PBS supplemented with 2%FBS and layered over Histopaque-1077(Sigma-Aldrich) to separate blood components from monocytes. The enriched monocyte layer was collected and washed with PBS. For monocyte-macrophage differentiation, 0.5 × 10^6^ monocytes (6-wells plates) and 0.2 × 10^6^ (24-well plates) were cultured and differentiated using 50 ng/mL of M-CSF (Immunotools) for 7 days in RPMI1640 medium, supplemented with FBS (10%v/v) and penicillin/streptomycin (1% v/v). Cells were maintained at 37 °C and 5% CO2 humidified atmosphere.

### CEA expression evaluation

CRC cell lines (HCT15, HCT116, HT29, RKO, SW480, SW48, Caco-2 and LS174T) were seeded in T25 cm^2^ flasks for 48 h. Cells were washed and incubated with accutase (Thermo Fischer) for 5 min at 37 °C and harvested. After, cells were washed and resuspended in FACS buffer (1% BSA in PBS). For staining it were used 0.05 × 10^6^ cells, anti-CEA monoclonal antibody (Invitrogen) diluted 1:100 in FACS buffer, primary antibody, Alexa 488 mouse, secondary antibody, diluted 1:200 in FACS buffer, and the Live/dead stain diluted 1:10,000 in PBS (ebioscience). Each antibody was incubated for 30 min at 4 ºC, followed by washing steps with FACS buffer. Cells were fixed with 2% of PFA, 15 min at RT, and washed and resuspended in FACS buffer. Samples were analyzed with a BD FACSCanto^™^ II flow cytometer (BD Biosciences). Unstained cells and single stained with antibodies were used as control. Median fluorescence intensity was measured for at least 10,000 viable plus single cell gated events per sample, and all data was processed with FlowJo software (Tree Star).

### Cell-nanoparticle interaction evaluations

Cellular uptake of anti-CEA-functionalized NPs (F NPs) and non-functionalized NPs (NF NPs) was quantified by imaging flow cytometry and evaluated qualitatively by confocal laser-scanning microscopy.

#### Imaging flow cytometry

To perform imaging flow cytometry analysis, 1 × 10^6^, or 0.5 × 10^6^ cells per well were seeded for Caco-2/SW48 and HCT15/SW480 cell lines (6-well plate), respectively. The cells were allowed to attach 24 h, after which media was replaced and 25 µgmL^ − 1^ of PLGA-FITC (Excitation maximum, nm: 490, Emission maximum, nm :520) labeled F NPs and NF NPs (safety concentration) and free anti-CEA scFv was added. Analysis was done at different time points,1,3, 10 and 24 h at 37 °C. Afterward, cells were washed twice with 500 µL of PBS and detached with 300 µL trypsin (Thermo Fischer) for 5 min. Cells were subsequently washed with PBS at 300 g for 5 min, fixed with 200 µL of 2% PFA for 20 min at RT, washed again, and resuspended in 80 µL of PBS for further analysis. Cells were analyzed quantified by imaging flow cytometry (ImageStreamX^®^, Amnis, EDM Millipore). FITC-NPs fluorescence was assessed by using a laser for excitation and images were acquired in channel 2 (490–560 nm) from the CCD camera using a 40 × objective. For brightfield images, a brightfield LED lamp was used, and images were collected on channel 1, 420–480 nm, and at least 150,000 events were collected. Image analysis was performed using IDEAS^®^ data analysis software (Amnis, EDM Millipore, version 6.2.64.0), following the Internalization wizard pipeline where the ratio intensity of the FITC-NPs fluorescence signal inside the cell to the intensity of the entire cell was quantified. To discriminate internalized versus membrane-associated NPs, we designed a mask for the whole cell, defined in the brightfield image (Channel 1), darkfield, side scatter (SSC), which provides information about the internal complexity (granularity) of a cell (channel 6) and a cytoplasmic mask (internal) performed by eroding the whole cell mask by 4 pixels. A control based only on cells without NPs was also performed to exclude the possible cellular autofluorescence.

#### Confocal laser-scanning microscopy

To evaluate the fate of F NPs on the cells, NPs were labelled with 30% of PLGA-Rhodamine B and compared with the control (PLGA- Rhodamine B labeled NF NPs). Briefly, CEA-expressing (Caco-2/SW48) and CEA-non expressing cell lines (HCT15/SW480) were plated in glass coverslips on 24-well plates (0.04 × 10^6^ cells per coverslip). Cells were left to adhere for 24 h. After, cells were washed with PBS and treated with 25 µg mL^ − 1^ of F NPs or NF NPs, for 1, 3, 10 and 24 h at 37 ºC. Cells were fixed with 4% PFA for 15 min at RT, and permeabilized with 0.1% Triton-X100 for 15 min at RT. For cell staining it was used DAPI (1:100) for nuclei, and Green Stain (HCS CellMask Green stain^™^) with initial concentration of 0.01 µg/mL (1:1000 dilution), for cell membrane, and Vectashield (Vector laboratories) used as mounting media. Cells were imaged with a SP5 confocal microscope (Leica Microsystems, Germany) and image analysis was performed with the LAS AF Lite software (Leica). Green fluorescence was obtained from the green channel (excitation wavelength: 493 nm, emission wavelength: 516 nm), representing the cell membrane and cytoplasm with HCS cell Mask. The blue fluorescence is from the blue channel (excitation wavelength: 365 nm, emission wavelength: 470 nm), which shows blue stained cell nucleic acid with DAPI. The red fluorescence is from the red channel (excitation wavelength: 633, emission wavelength: 690 nm), which shows the red-stained of F NPs and NF NPs with rhodamine B.

### Cell metabolic activity evaluation

Resazurin assay was used to analyze cell metabolic activity. Therefore, 0.002 × 10^6^ cells of Caco-2/SW48 and 0.004 × 10^6^ cells of HCT15/SW480 were seeded in 96-well plates and allowed to attach during 24 h. 2 × 10^5^ of monocytes, were differentiated in 24 well plates for 7 days, as described above. Subsequently, in both, the medium was removed, cells were washed with PBS, and different concentrations of free 5-FU or NPs (2.5, 5, 10, 50, 75, 100, 500 µM regarding the drug) in medium (200 µL) were incubated for 24 h, 48 h and 72 h, at 37 °C. NH_4_OH was added as a negative control and cells without treatment as a positive control. At each timepoint, resazurin redox dye (0.01 mg/mL, Sigma-Aldrich) was added (1/10 of the total volume of culture medium) to cell culture, incubated for 4 h at 37 °C and 5% CO_2_, and the fluorescence intensity was measured at 530/590 nm using a SynergyMx MultiMode microplate reader (BioTek). All data were normalized using, background medium, negative and positive (cells without treatment) controls, which were considered 0% and 100% metabolic activity, respectively. The cell metabolic activity was analyzed using as a threshold of 70% cell viability according to the ISO 10993–5 standard [28].

### Impact of NPs on macrophage polarization

As described above, 0.5 × 10^6^ monocytes were plated in 6-well plates and differentiated into macrophages. After 7 days of differentiation, RPMI 1640 medium was removed, and macrophages were washed. As a controls of polarization, macrophages were incubated with 10 ng/mL of LPs (M1- like) or 10 ng/mL of IL-10 (M2—like), for 4 h. M0 macrophages, were incubated without any addiction of exogenous factors. After, 75 µM of free drug and NPs (in regard of drug concentration) were added for 72 h. To evaluate macrophage polarization, macrophages were detached with accutase at 37 °C during 30 min and harvested by gently scrapping. Cells were washed and resuspended in FACS buffer containing appropriate conjugated antibodies, and stained in the dark for 40 min at 4 °C. Briefly, macrophages were stained with the following antibodies: anti-human CD14/APC (ref 620926) (1:50), anti-HLA-DR/PB (ref 2437643) (1:250), anti-CD86/FITC (ref 277175) (1:50), anti-CD206/PE (ref B353712) (1:50), anti-CD163/PE (ref. 1260877) (1:50) and anti-PDL1-FITC (ref.1055403) (1:50). After 40 min of incubation, cells were washed twice and incubated with Live/Dead stain (1:10,000) in PBS for 30 min at 4ºC. Cells were washed twice and fixed with 2% PFA for 15 min at RT, washed again, and placed into cytometer tubes for further analysis. Samples were analyzed with a BD FACSCanto™ II flow cytometer (BD Biosciences). To define background, unstained controls were used and to compensate staining overlaps beads-matched antibodies were used. Median fluorescence intensity was measured for at least 50,000 viable plus single cell gated events per sample, and all data was processed with the FlowJo software (Tree Star). All experiments were performed, at least, in triplicate.

## Statistical analysis

Results were expressed as mean  ± standard deviations (SD) of minimum three independent experiments. Data was analyzed using Student's t-test for comparison between two independents groups and ANOVA for comparison among three or more groups, in GraphPad Prism 8.0 software (GraphPad, USA). The level of significance was set at probabilities of ∗ p  < 0.05; ∗  ∗ p  < 0.01 and ∗  ∗  ∗  ∗ p  < 0.001.

## Results

### Chemical synthesis of Anti-CEA scFv with PLGA-PEG-mal polymer

**Fig. 1:**
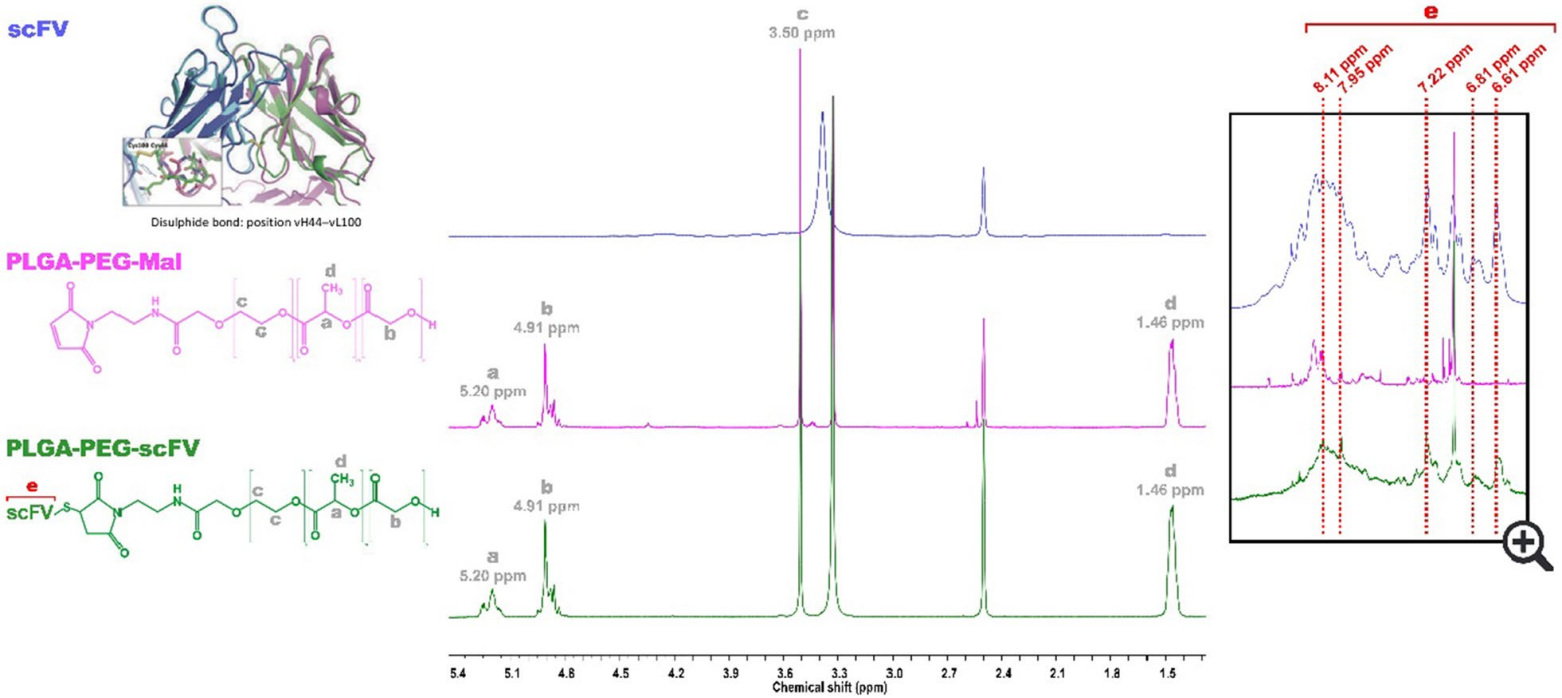
^1^H NMR analysis of the chemical conjugation between anti-CEA scFv and PLGA-PEG co-polymer.^1^H NMR characterization of PLGA-PEG-anti CEA scFv polymer: characteristic peaks of **a**, **d** glycolic acid, **b** lactic acid; **c** PEG and **e** anti-CEA scFv in the final PLGA-PEG—scFv polymer (right)

The sulfhydryl group of the anti-CEA scFv, MFE-23, was chemically conjugated with the maleimide group of the PLGA-PEG-Mal polymer to obtain the CEA-targeted PLGA-PEG-scFv polymeric conjugate [24, 29]. A PLGA-PEG-Mal polymer containing a PEG of 5 K was selected since this molecular weight has been associated with a prolonged blood circulation time due to lower absorption of serum proteins and interaction with macrophages reduced kidney’s clearance, therefore increasing the probability of a successful anti-tumor therapy [30, 31]. The conjugation protocol herein used was previously optimized by our group [32, 33], allowing us to obtain an anti-CEA scFv conjugation efficiency (CE) of around 90%. Additionally, ^1^H NMR confirmed the successful reaction between the anti-CEA scFv and PLGA-PEG-Mal, revealing characteristics peaks of the polymer (δ = 5.20, 4.91, 3.50 and 1.46 ppm) [32, 33] and of the scFv (δ = 8.11, 7.95, 7.22, 6.81, 6.61 ppm) [34] in the spectrum of the final polymeric conjugate (Fig. 1).

## Manufacture and characterization of 5-FU-loaded CEA-targeted NPs

**Fig. 2:**
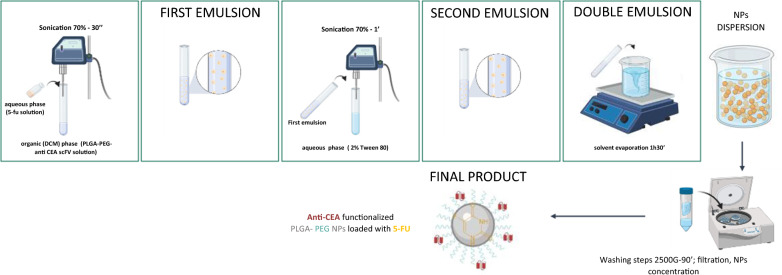
Schematic illustration of the methodology of nanoparticles production

Despite the clinical significance of 5-FU, its systemic toxicity, low bioavailability, short half-life, and frequent drug resistance, are still limiting its therapeutic effect. Therefore, to overcome these drawbacks, the development of sustained release formulations of 5-FU such as NP systems may have an important impact in the improvement of the therapeutic response, providing effective tumor regression with minimal side effects in comparison with the native form of the drug [35]. However, 5-FU encapsulation into NP systems is characterized by several drawbacks, including the drug’s hydrophilic properties and small size (MW 130.08 Da). Consequently, the challenging weak interactions with NP system matrices such as PLGA, lead to low encapsulation efficiency, undesired leakage, and initial burst release [36]. In this work, 5-FU was encapsulated in a functionalized anti-CEA targeted PLGA-PEG-scFv NPs, (F NPs), by double emulsion technique (Fig. 2), achieving a drug loading (DL) of 8.3 ± 3.85% (Table 1). This finding is in agreement with the solubility profile of 5-FU as well as its hydrophilicity which leads to 5-FU diffusion to the aqueous external phase during the encapsulation process [37].

**Table 1:** Physicochemical properties of functionalized and 5-FU loaded nanoparticles as well as respective controls, with mean size (Z-average size), polydispersity index, surface charge (ζ-Potential), and DL (%). Values are represented as mean values ​ ± ​SD (N  = 6). Non applicable (−)

	Size (nm)	Polydispersity index	Surface charge (Mv)	Drug loading (%)
Empty PLGA NPs	164.6 ± 5.1	0.21 ± 0.01	− 7.46 ± 0.30	
5-FU loaded PLGA NPs	169.9 ± 2.9	0.19 ± 0.01	− 6.62 ± 0.38	4.7 ± 2.3
Empty NF NPs	142.4 ± 6.8	0.25 ± 0.03	− 5.55 ± 0.81	
Empty N NPs	136.8 ± 2.1	0.22 ± 0.01	− 9.22 ± 0.84	
5-FU loaded F NPs	133.7 ± 1.3	0.25 ± 0.01	12.53 ± 0.37	8.3 ± 3.9

Regarding the physicochemical properties of NPs, Z-average size of 5-FU-loaded F NPs and respective controls ranged from approximately 130 and 170 nm, with a relatively monodisperse size distribution characterized by a polydispersity index of around 0.2. Additionally, a decreasing ζ-Potential of nanoparticles with scFv was observed due to the net negative charge on their surface from the scFv moiety [38].

**Fig. 3:**
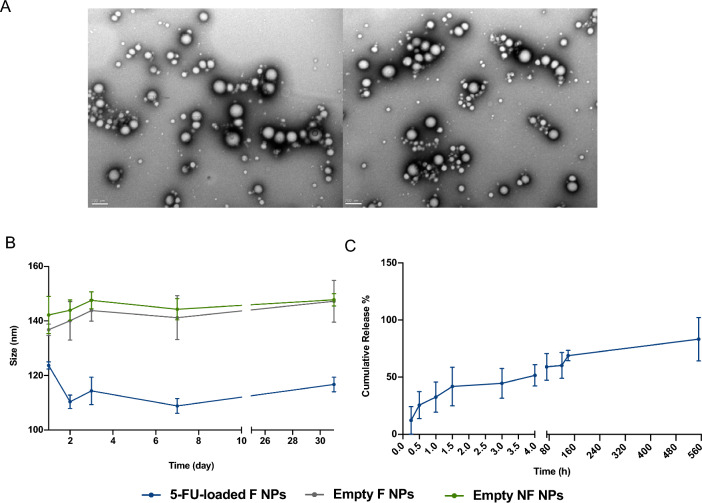
Nanoparticles physicochemical characterization and in vitro release of 5-FU. **A** Image of empty F NPs (left) and of 5-FU-loaded F NPs (right) obtained by Transmission Electron Microscopy (TEM). Scale bar: 200 nm. **B** Stability of empty nanoparticles (NF NPs), functionalized unloaded NPs (empty F NPs) and of 5-FU-loaded F NPs, in cell culture medium at 4 °C for 1 month. **C** in vitro release profile of 5-FU from F NPs in PBS pH 7.4 at 37 °C during 552h. **C**. All measurements were done in triplicate and results are presented as mean ± SD

Furthermore, NPs revealed a spherical shape with relatively smooth surfaces and uniform size distribution in all samples as shown through TEM analysis. The nanoparticles stability was assessed over 1 month with a relatively constant size (Fig. 3A, B). Moreover, the 5-FU cumulative release had 2 phases (Fig. 3C): (i) an initial burst release, in accordance of described for several authors due to the initial dissolution of entrapped drug attached to the NPs surface, and some particular properties of the nano system, such size, porosity, MW and drug hydrophilicity [39]; and (ii) a second release step, with slow drug diffusion from the PLGA matrix, being associated with the erosion and/or swelling of PLGA in release medium [40, 41]. Nevertheless, these data were obtained from an in vitro mimicking analytical assay which may not allow extrapolation of conclusions regarding the initial burst release in in vivo conditions.

## CEA expression analysis in colorectal cancer cell lines

To assure that the develop NPs could hold maximum specificity, a full screening of CEA expression in a number of CRC cell lines from different CMSs was performed by flow cytometry. The obtained results showed different levels of CEA expression, with lower expression levels in SW480, HCT-15, HCT-116 and RKO cell lines. The higher values were achieved in Caco-2, HT-29, LS174T and SW48 cell lines, though from different CMSs (Additional file 1: Figures S1, S2). These findings are in concordance with recent reports referring that, despite the transversal CEA expression in CRC stages, CRC’s intratumor and intertumoral heterogenicity with different activated molecular pathways leads to distinctive CEA levels of plasticity and expression [42] Additionally, the efficacy of 5-FU treatments is being correlated with CMSs and molecular subtypes [43]. Therefore, to conduct NPs efficacy studies in different CMS and (MSI or MSS, MSI cell lines from CMS1, representing the CRC subpopulation where 5-FU has less efficacy and immunotherapy is illegible, were selected as CEA ^high^ (SW48) and CEA ^low^ (HCT-15). Moreover, MSS cell lines from CMS4, representing the CRC subpopulation with inactivated immune infiltrate, immunosuppressive TME and where 5-FU has been correlated with no significance in OS, were also selected and included cell lines CEA ^high^ (Caco-2) and CEA ^low^ (SW480). The selection of each cell line, categorized as MSI or MSS in the respective CMS, had in consideration the work published by Berg K. et al. [44].Additional file: As per journal requirements, every additional file must have a corresponding caption. In this regard, please be informed that the caption of Additional file [1] was taken from the additional e-file itself. Please advise if action taken appropriate and amend if necessary.ok

## Targeting capacity of anti-CEA scFv functionalized NPs towards CEA^high^ and CEA^low^ CRC cells

**Fig. 4:**
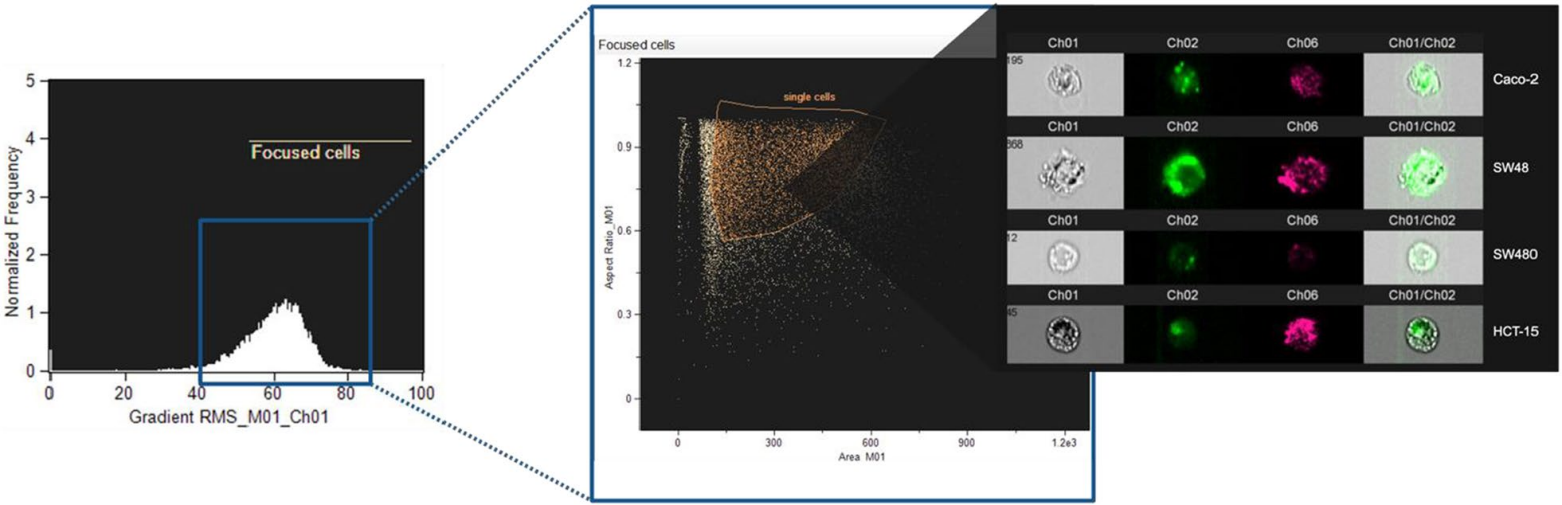
Gating strategy used to select cells in focus and separate them from the doublets and non-focused events in untreated cells (left image). Gating strategy for single cells from focused cells in the middle. Features for the bivariate dot plot are calculated based on a Brightfield image and using the mask that covers the whole cell (channel 1, ch1). Proof of successful separation of the cells from the non-focused and doublets verified in imaging gallery with respective fluorescence in each channel (Caco-2; SW48; SW480 and HCT-15 cell lines incubated with F NPs along 24 h) (right image)

To evaluate the specificity and selectivity of the developed CEA-targeted NPs, interaction with the aforementioned CRC cell lines was investigated. Therefore, to understand the impact of the targeted strategy on cell-NP interaction, a protocol for image flow cytometry evaluation was established with prior detachment of the cells with trypsin, that allowed to remove the majority of non-internalized NPs, present on the surface of the cells and have accurate results. To infer their specificity to target cells, the ImageStream flow cytometry technology was used, since it allows simultaneous evaluation of a given molecule of interest expression and localization, allowing the acquisition of multiple images of each cell in flow. Brightfield images were recorded to differentiate cell membrane and cytoplasm (Channel 1). NPs were tracked in Channel 2, where significant differences in the degree of cellular interaction between empty non-functionalized (NF NPs) and CEA-targeted NPs (F NPs) were observed in CEA-expressing cells (Fig. 4). In addition, channel 6 provided information about the granularity of the cells (internal complexity). Unstained cells were included as controls (brightfield in channel 1 and darkfield (SSC) in channel 6).

**Fig. 5:**
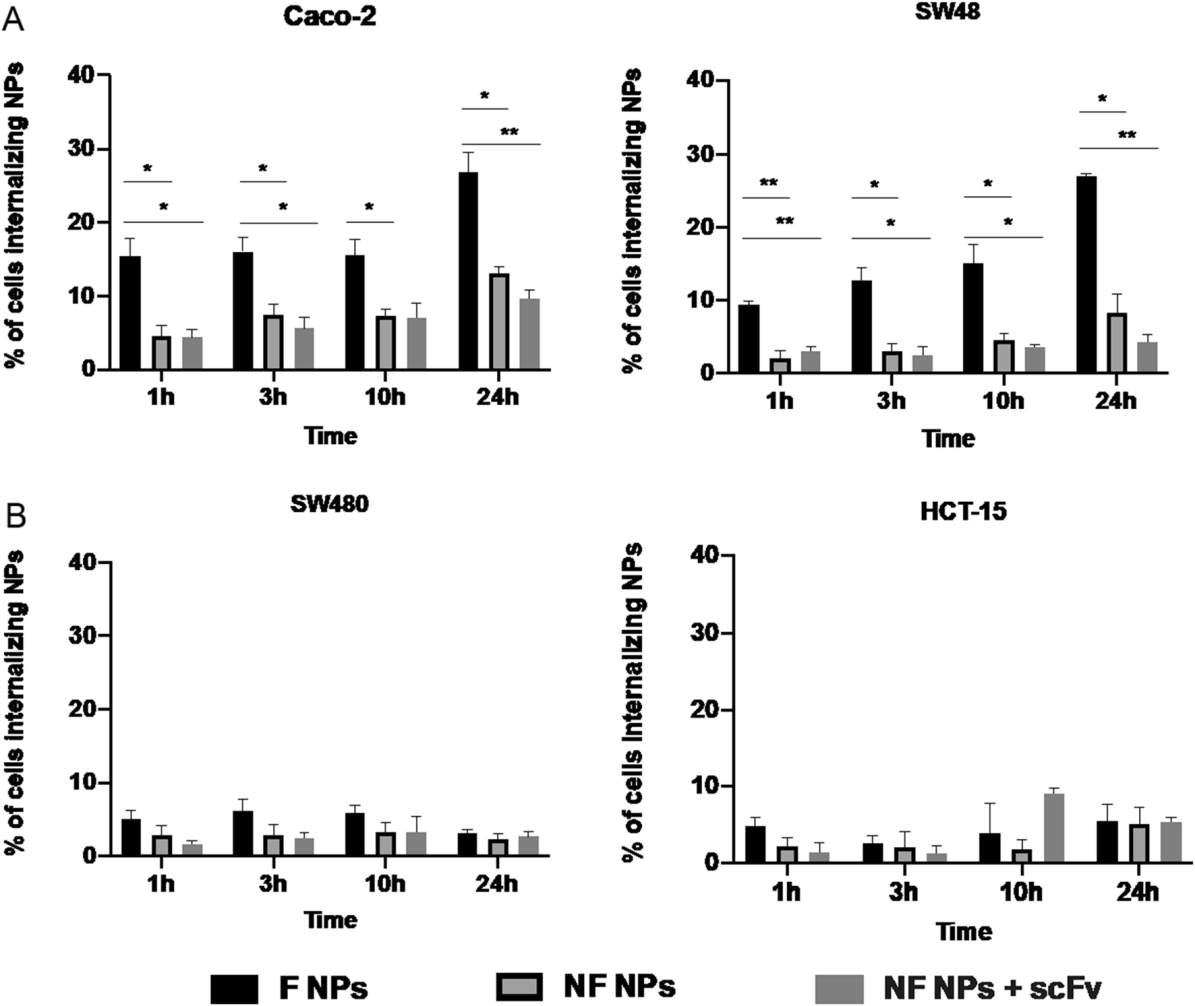
Imaging Flow cytometry analysis of cell internalization of F and NF NPs or NF NPs incubated with free anti-CEA (NF NPs + scFv) into A) CEA ^high^ cell lines (Caco-2 MSS cell line on the left and Sw48 MSI cell line on the right) and into B) CEA-non expressing cell lines (SW480, MSS cell line, on the left and, HCT-15, MSI cell line, on the right). Expression values were normalized to the unstained control. Statistical comparison of functionalized with non-functionalized NPs. *p < 0.05, **p < 0.01, ***p < 0.001 or ****p < 0.0001

The F NPs showed 2–threefold higher internalization in CEA^high^ expressing MSS and MSI CRC cell lines, indicating the specificity of the developed targeted nanosystem. As shown in Fig. 5A, Caco-2 cell line (MSS CEA^high^ cell line) presented a significant increase in the internalization rate of F NPs comparing to NF NPs (*p* = 0.0106, *p* = 0.0320, *p* = 0.0350 and *p* = 0.0411), along the different times of incubation (1, 3, 10 and 24 h), respectively. Importantly, the incubation of NF NPs with the free anti-CEA scFv (NF NPs + scFv) did not result in an improvement of internalization rate by the Caco-2 cell line, highlighting the importance of anti-CEA scFv functionalization on the surface of NPs. Additionally, equivalent results were found in the SW48 cell line (MSI CEA^high^ cell line) with critical differences between F NPs and NF NPs as well as F NPs and NF NPs incubated with free scFv at time point 1 h (p = 0.0056 and p = 0.0067) respectively. Moreover, changes in the internalization rate of F NPs vs NF NPs were maintained along 24 h of incubation (*p* values of 0.0283 for 3 h, 0.0278 for 10 h and 0.0142 for 24 h). Similar results comparing F NPS vs NF NPS + scFv from 3 h, 10 h and 24 h of incubation (p = 0.0308; 0.0330 and < 0.0001, respectively) were obtained. The higher cell internalization of F NPs compared to NF NPs was reflected by an increase of up to three times in the percentage of focused positive cells, over 24 h (Fig. 4). In fact, our results corroborated the previously reported tumor cell specificity of the anti-CEA scFv, when used to functionalize Superparamagnetic iron oxide nanoparticles [22, 45]. In addition, we had also confirmed the CEA-targeting specificity of our system by evaluating the same NPs conditions in MSI and MSS CEA^low^ expressing CRC cell lines. The results presented non-significant changes between NP testing conditions (Fig. 5B), being the cellular uptake independent on the presence of the anti-CEA scFv in the NPs surface. Therefore, our results confirmed the specific targeting of CEA-targeted NPs for MSI/MSS CEA^high^.

To improve our knowledge about a possible intracellular localization of the developed NPs, we examined the fluorescent signal of F NPs and NF NPs after incubation with MSI and MSS CEA^high^ and CEA^low^ CRC cells during 24 h, using confocal microscopy (Additional file 1: Figure S3). F NPs were observed to have a much higher cell affinity to Caco-2 and Sw48 cells (CEA^high^) than NF NPs, which did not demonstrate an evident interaction with these cancer cells. Moreover, we observed that some of the NF NPs pools were localized outside the cell membrane (Additional file 1: Figure S3B, S3C), while F NPs were effectively found inside the cells, as demonstrated through overlay’s orthogonal projections in XZ and YZ in SW48 cells (Additional file 1: Figure S3D). In fact, in solid tumors, high-affinity antibodies have been described to bind to the antigen of interest, but their lengths affect their retention at the tumor cell surface. In particular, CEA was reported as a non-internalizing antigen [46]. However, recent studies suggested CEA as an internalizing antigen, being the anti-CEA antibody penetration into the cells caused by the metabolic turnover of the CEA of around 16 h and associated with alternative transport pathways not involving clathrin [47, 48]. Therefore, since the internalization of anti-CEA antibodies is reported as faster than receptor-mediated endocytosis, the alternative endocytic pathway may involve a similar mechanism of non-clathrin-coated vesicle pathway [49]. Moreover, as presented in Fig. 5, when comparing F NPs with NF NPs + scFv, F NPs had no evidence of scFv absorption to NF NPs. Hence, our data reinforced the potential of CEA as a promising target for anti-CRC therapies, favoring the internalization of drug-loaded NP systems.

## Effect of 5-FU-loaded CEA-targeted NPs in the metabolic activity of CRC cells

**Fig. 6:**
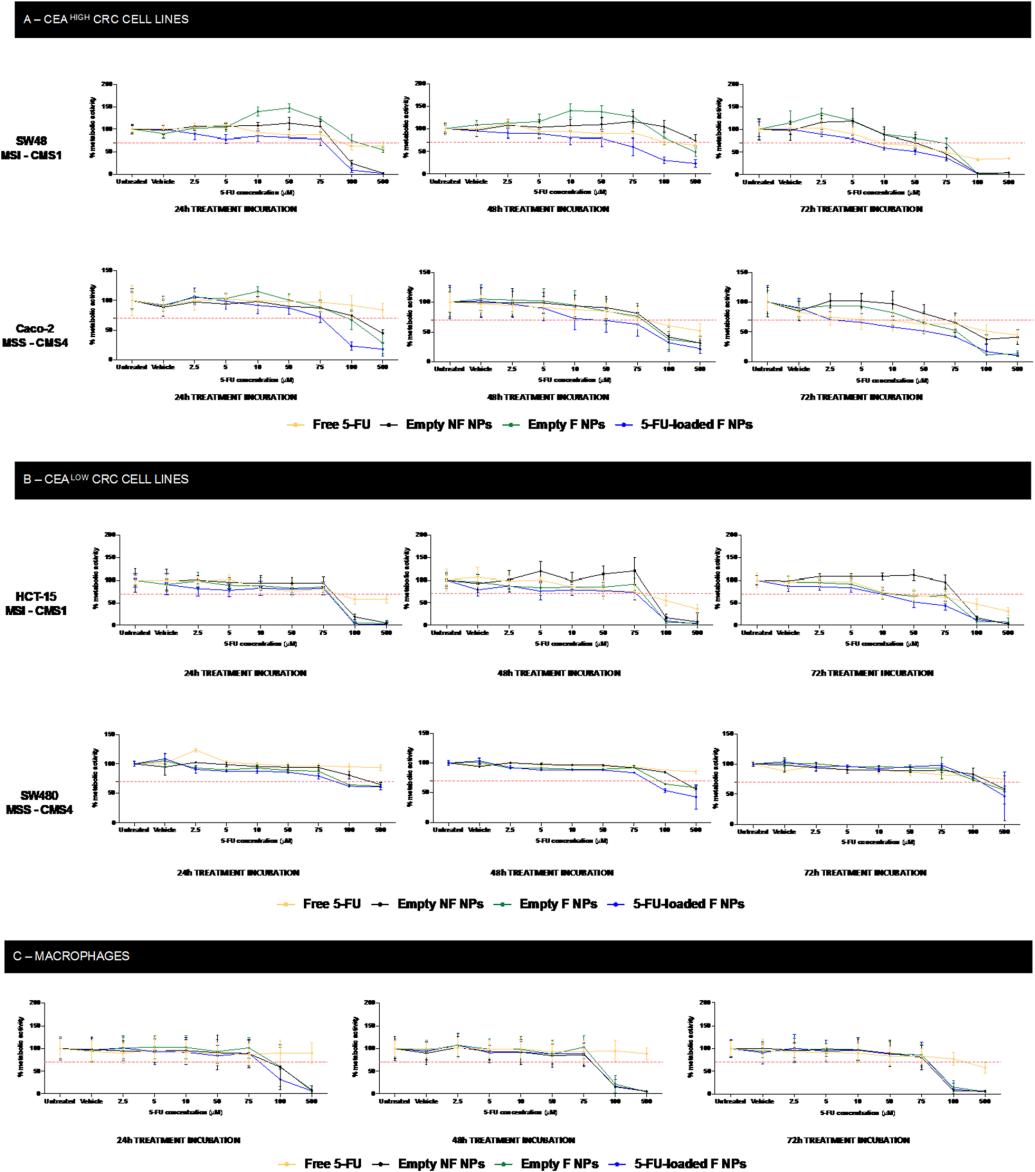
Evaluation of metabolic activity in CRC MSI and MSS cell lines when incubated with free 5-FU, empty NF NPs, F NPs and 5-FU loaded F NPs at 24 h, 48 h and 72 h. **A** Metabolic activity assessment of CEA ^high^ cells. **B** Cell viability evaluation in CEA ^low^ cells. **C** Macrophages metabolic activity. Statistical comparison of 5FU-loaded F NPs with the free drug 5-FU, within each dose range. *p < 0.05, **p < 0.01, ***p < 0.001 or ****p < 0.0001

The ability of the developed NPs to compromise the metabolic activity of CRC cells was assessed in vitro through resazurin assays in the selected CEA ^high^ and CEA ^low^ CRC cell lines (Fig. 6). The metabolic activity was evaluated over 24 h, 48 h and 72 h using 5-FU doses ranging from 2.5 to 500 µM. For both CEA ^high^ CRC cell lines (Caco-2 and SW48), the metabolic activity of the cells treated with 5-FU-loaded anti-CEA functionalized NPs (5-FU loaded F NPs) significantly decreased when compared with the free 5-FU, after 24 h of incubation. Moreover, in SW48 cells, the reduction on the metabolic activity was significantly affected from 5 µM (*p* < 0.0001) when compared with the 5-FU free drug, after 24 h. Over 48 h, the metabolic activity was only impacted from 75 µM (*p* = 0.0086). Moreover, the respective controls, consisting of unloaded NF NPs and F NPs, were considered safe until 75 µM after 48 h, and until 50 µM after 72 h with above 70% of viable cells [28]. Despite the improvements in the reduction of cell metabolic activity from 10 µM in 24 h, in CRC CEA ^high^ MSS—CMS4 cell line, Caco-2, the statistical significance was only reflected from 75 µM (p < 0.0001). In similarity with SW48 cells, the controls using unloaded NF NPs and F NPs showed safety until 75 µM in 48 h. No significant values of lower metabolic activity, indicating cell death, were found at 72 h. These findings revealed the capability of the targeted NPs to induce higher cell death rates in CRC CEA-expressing MSI and MSS cells within 24 h, in contrast with free 5-FU, which takes 72 h to impact cancer cell in the same way (IC50 = 75 µM) (Fig. 6B) [50–52]. Accordingly, treatments in the same range of concentrations were added to the selected CEA ^low^ MSS and MSI cell lines and, despite the lower expression of CEA, the nanosystems showed better pharmacological performances with a significant impact in cell metabolic activity in 48 h. For SW480, MSS cell line p < 0.001 as found from 2.5 µM to 500 µM. Moreover, despite the reduction in cell viability in HCT-15, MSI cell line, no significant statistical values were found until 75 µM (p < 0.0001 for 100 and 500 µM). Additionally, in both CEA ^low^ cell lines, unloaded NF NPs and F NPs exhibited safety until 75 µM after 72 h (Fig. 6C). The same experiment was done in primary human macrophages, isolated from healthy blood donors. No differences in macrophages metabolic activity were obtained until 75 µM after 72 h (Fig. 6D).

According to the literature, the IC50 of ~ 60 µM and ~ 300 µM for free 5-FU drug, after 72 h of incubation with SW48 and SW480 5-FU resistant cell lines, respectively, are in accordance with our findings, suggesting that our cells lines spontaneously generated 5-FU resistance as described by other authors [50, 52]. Moreover, similar IC50 ~ 10 µM for free 5-FU drug and 5-FU loaded F NPs incubation with Caco-2 and HCT15 5-FU sensitive cell lines was found in this work [51, 53]. Therefore, the obtained results agreed with the reported IC50 for 5-FU free drug, showing a potent efficacy of the 5-FU-loaded F NPs within 24 h of incubation with CEA ^high^ cell lines, in comparison with the free drug. Additionally, reported studies are describing an enhancing cytotoxic potential when NPs were surface modified with a targeting ligand [19], as well as an increase of toxic effects when polymeric nanoparticles repeated-dose exposure, inducing a certain osmotic pressure on the cells, which may justify our results [54].

## Impact of 5-FU-loaded F NPs on the polarization of macrophages

TAMs are described as controversial in CRC progression and treatment response prediction, according to their polarization status [55]. CD206^+^ TAMs have been positively correlated with recurrence-free interval duration [56] and described as able to mediate resistance to 5-FU chemotherapy [9]. Here, we evaluated the potential impact of the developed NPs on macrophage polarization, since 5-FU-based chemotherapy has been reported as a critical factor for macrophages polarization, consequently resulting in divergent treatment responses.

**Fig. 7:**
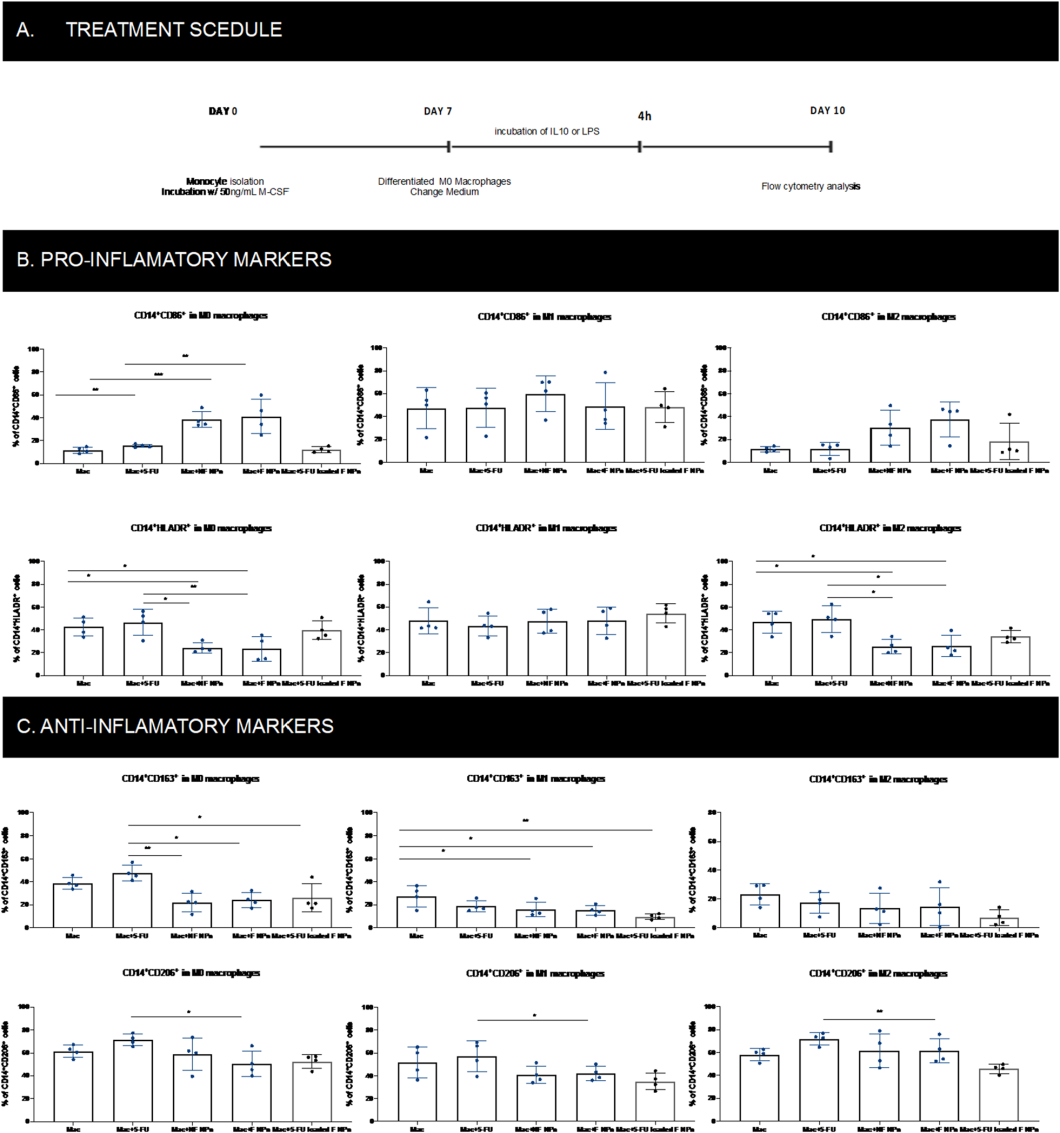
Impact of NPs on macrophages polarization. **A** Experiment schedule **B** Percentage of cells expressing pro-inflammatory markers (CD86, HLA-DR) on unstimulated, M1-like and M2-like macrophages surface **B** Percentage of cells expressing anti -inflammatory markers (CD163, CD206) on unstimulated, M1-like and M2-like macrophages surface

Experimentally, we first stimulated macrophages with IL-10 or LPS to confirm their polarization capacity into a M2 or M1-like macrophages, respectively. Their phenotype was evaluated by the surface expression of CD86 and CD163 as M1 and M2 markers, respectively, confirming their capacity to polarize when compared with the non-stimulated macrophages (Fig. 7). As expected, LPS stimulation decreased the expression of CD163^+^ cells and increased the expression of CD86^+^ cells, while IL-10 stimulation had the opposite effect.

To determine whether unloaded NF NPs, F NPs and 5-FU-loaded F NPs had a similar impact of free 5-FU on macrophages polarization, 75 µM of NPs (safe concentration observed above) was added to unstimulated, LPS or IL-10-stimulated macrophages. When unstimulated macrophages were incubated with NF NPs, the percentage of CD86^+^ cells increased, whereas the percentage of HLA-DR + cells decreased (p < 0.01, Fig. 7B). However, when the anti-inflammatory surface markers (CD163 and CD206) were evaluated, no significant changes were observed. Additionally, when compared with the administration of free 5-FU drug, the administration of NF NPs could sustain the initial levels of untreated and unstimulated macrophages (Fig. 7C). When macrophages were stimulated with LPS, acquiring a M1-like phenotype, no changes in the percentage of cells expressing pro-inflammatory markers (CD86 and HLA-DR) were found, despite the tendency to decrease the percentage of cells expressing anti-inflammatory markers (CD163 and CD206), comparing with free 5-FU treated macrophages (Fig. 7B, C). Lastly, in the M2- like macrophages stimulated with IL-10, a slight decrease of HLA-DR expression levels was achieved but with no significant differences in the percentage of CD86^+^ expressing cells. No changes were obtained in the percentage of cells expressing the anti-inflammatory markers CD163 and CD206. Moreover, in unstimulated and LPS-stimulated macrophages, a tendency in keeping the initial expression of CD206 surface levels was observed when compared with the impact of free 5-FU in this marker.

Overall, no significant changes were achieved on the macrophages’ polarization, despite the tendency to maintain the initial expression of surface markers in untreated macrophages. Moreover, the immunogenicity of associated antibody fragments shown no effect on macrophages, proving a safe administration of the developed NPs.

## Conclusions

We developed an innovative nanoplatform to deliver 5-FU to CRC, based on the functionalization of a PLGA and PEG polymer with an anti-CEA scFv, namely MFE-23. To our best knowledge, this is the first proof-of-concept using an anti-CEA scFv chemically conjugated with PLGA-PEG polymers for nano-chemotherapeutic applications, facilitating the delivery of a conventional drug to treat CRC. Here, we observed the ability of CEA-targeted NPs to provide a specific targeting to CEA-expressing cells with 3-times enhanced rates of internalization when compared with NF NPs. Furthermore, the developed strategy was able to significantly decrease the metabolic activity of CEA-expressing cells in 24 h and 48 h, conferring better anti-cancer activity to the delivered drug. Moreover, the CEA-targeted NPs loaded with 5-FU were considered safe for macrophages, with no relevant biological impact on macrophages polarization, proving the suitability of the strategy for further application in a more complex 3D model with associated TAMs mimicking the surrounding TME. The developed therapy might bring a new perspective of investigation to explore the advanced delivery system with higher loading capacity and minimum adverse effects on CEA-expressing CRC tumors. Additionally, CEA-targeted NPs may also offer an opportunity for further targeted therapy applied to different types of cancer and metastatic sites of CRC, normally expressing CEA. Altogether, we developed a targeted strategy that could constitute a new outlook of chemotherapy employment in association with combinatory immunotherapeutic strategies.

### Supplementary Information

**Additional file 1**: ** Figure S1.** Evaluation of surface CEA expression levels in live CRC cell lines. **A** percentage of CEA positive cells within live cell, analyzed by flow cytometry. **B** Gating strategy applied during the analysis in Flow Jo software The scatter plots exhibit a representative image of the gating strategy created with FlowJo software for flow cytometry analysis. FSC-A/SSC-A exemplifies the distribution of cells in the light scatter based on cell size and granularity, respectively; FSC-A/FSC-H represents the single cells of the previously selected population. **Figure S2.** Expression levels of CEA, determined by flow cytometry in the selected MSS and MSI CRC cell lines and their subclassification according to CMS. The control is presenting the autofluorescence of cells in each respective unstained selected cells. **Figure S3.**. Confocal microscopy analysis of cell internalization of NF NPs and F NPS into same cell lines at 24 h of incubation. **A** NPs are stained in red. **B** Green Cell mask was used to stain and define the cytoplasm cell area, nucleus are stained in blue. Therefore, **B** is representing F NPS and NF NPs interaction with Caco-2 cells and **C** with SW480 cells. The red arrows point to the NPs. **D** 3D projection is reproducing the overlap between XZ and XY axis, evidencing F NPs inside Sw48 CEA ^high^ Cells. Scale bars represent 60 µm

## Data Availability

The collected and analyzed datasets during this study are available from the corresponding author on reasonable request.
