# Supplementary material for: scFv biofunctionalized nanoparticles to effective and safe targeting of CEA-expressing colorectal cancer cells
Source: J Nanobiotechnology. 2023 Oct 2;21:357. doi: 10.1186/s12951-023-02126-4 (PMC10544461; doi:10.1186/s12951-023-02126-4)
Supplement: Supplementary file 1 — Additional file 1: Figure S1. Evaluation of surface CEA expression levels in live CRC cell lines. A percentage of CEA positive cells within live cell, analyzed by flow cytometry. B Gating strategy applied during the analysis in Flow Jo software The scatter plots exhibit a representative image of the gating strategy created with FlowJo software for flow cytometry analysis. FSC-A/SSC-A exemplifies the distribution of cells in the light scatter based on cell size and granularity, respectively; FSC-A/FSC-H represents the single cells of the previously selected population. Figure S2. Expression levels of CEA, determined by flow cytometry in the selected MSS and MSI CRC cell lines and their subclassification according to CMS. The control is presenting the autofluorescence of cells in each respective unstained selected cells. Figure S3.. Confocal microscopy analysis of cell internalization of NF NPs and F NPS into same cell lines at 24 h of incubation. A NPs are stained in red. B Green Cell mask was used to stain and define the cytoplasm cell area, nucleus are stained in blue. Therefore, B is representing F NPS and NF NPs interaction with Caco-2 cells and C with SW480 cells. The red arrows point to the NPs. D 3D projection is reproducing the overlap between XZ and XY axis, evidencing F NPs inside Sw48 CEA high Cells. Scale bars represent 60 µm [file 12951_2023_2126_MOESM1_ESM.docx]

**Additional file information**

Figure S1. Evaluation of surface CEA expression levels in live CRC cell lines. A) percentage of CEA positive cells within live cell, analyzed by flow cytometry. B) Gating strategy applied during the analysis in Flow Jo software The scatter plots exhibit a representative image of the gating strategy created with FlowJo software for flow cytometry analysis. FSC-A/SSC-A exemplifies the distribution of cells in the light scatter based on cell size and granularity, respectively; FSC-A/FSC-H represents the single cells of the previously selected population.

Figure S 2. Expression levels of CEA, determined by flow cytometry in the selected MSS and MSI CRC cell lines and their subclassification according to CMS. The control is presenting the autofluorescence of cells in each respective unstained selected cells.

**
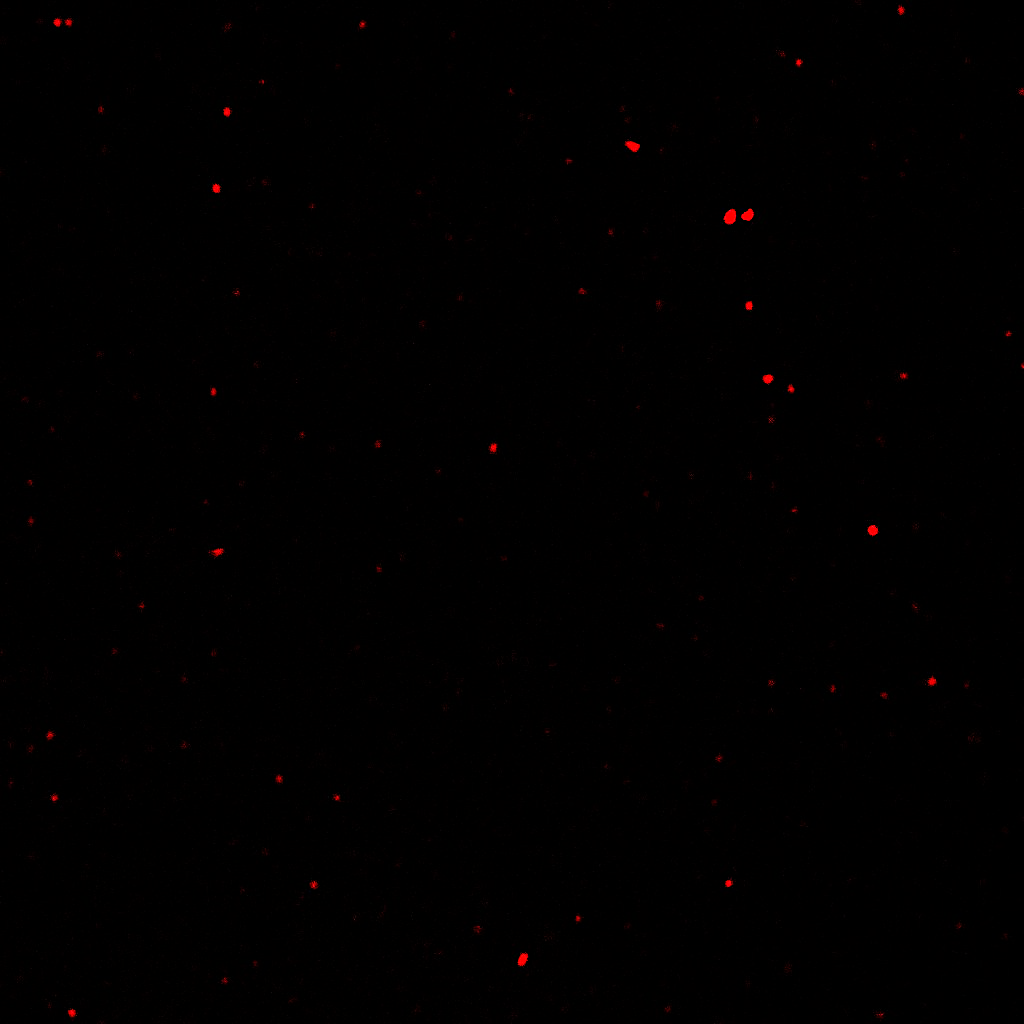
**

A

D

**
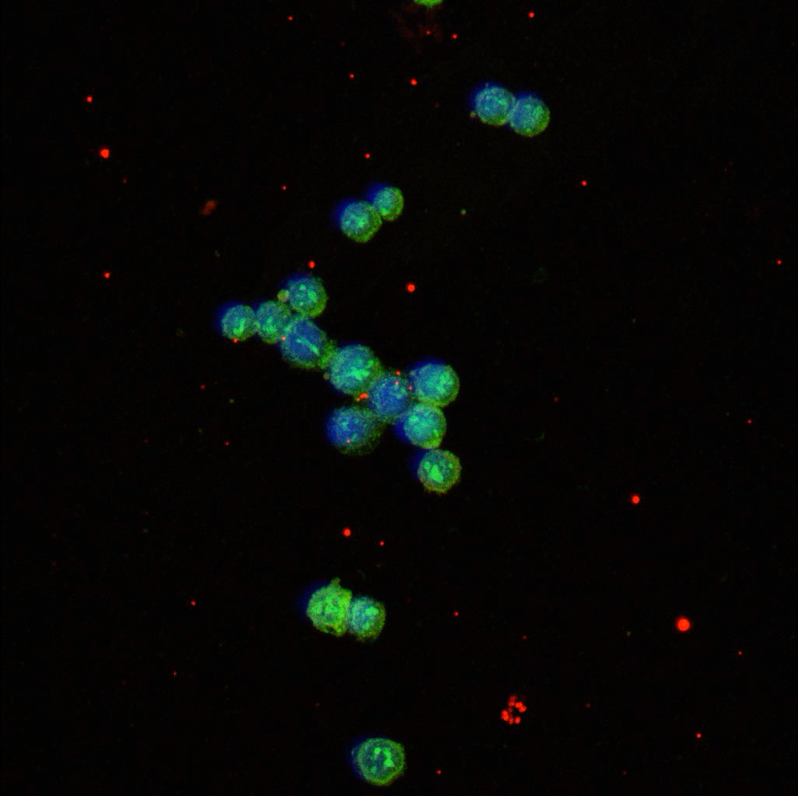
**

D

Figure S 3. Confocal microscopy analysis of cell internalization of NF NPs and F NPS into same cell lines at 24h of incubation. A) NPs are stained in red. B) Green Cell mask was used to stain and define the cytoplasm cell area, nucleus are stained in blue. Therefore, (B) is representing F NPS and NF NPs interaction with Caco-2 cells and (C) with SW480 cells. The red arrows point to the NPs. D) 3D projection is reproducing the overlap between XZ and XY axis, evidencing F NPs inside Sw48 CEA ^high^ Cells. Scale bars represent 60 µm.
